# The association between education and cardiovascular disease incidence is mediated by hypertension, diabetes, and body mass index

**DOI:** 10.1038/s41598-017-10775-3

**Published:** 2017-09-28

**Authors:** Irene R. Dégano, Jaume Marrugat, Maria Grau, Betlem Salvador-González, Rafel Ramos, Alberto Zamora, Ruth Martí, Roberto Elosua

**Affiliations:** 1 CIBER of Cardiovascular Diseases (CIBERCV), ISCIII, Madrid, Spain; 2 REGICOR Study Group. Cardiovascular Epidemiology and Genetics Group. Epidemiology and Public Health Program. IMIM (Hospital del Mar Medical Research Institute), Barcelona, Spain; 3 School of Pharmacy, University of Barcelona, Barcelona, Spain; 4 ABS Florida Sud and Cardiovascular Research Group in Primary Care (MACAP Costa Ponent), Primary Care Research Institute Jordi Gol, Catalan Institute of Health, l’Hospitalet de Llobregat, Barcelona, Spain; 5 Vascular Health Research Group (ISV-Girona). Institut Universitari d’Investigació en Atenció Primària Jordi Gol (IDIAP Jordi Gol), Barcelona, Spain; 6 Translab Research Group, Department of Medical Sciences, School of Medicine, University of Girona, Girona, Spain; 7 Institut Català de la Salut, Àmbit d’atenció Primaria, Girona, Spain; 8 Lipid and Atherosclerosis Unit and Department of Internal Medicine, Hospital de Blanes, Girona, Spain; 9 Institut d’Investigació Biomèdica de Girona (IDIBGI), Hospital Universitari Dr. Josep Trueta, Girona, Spain

## Abstract

Education and cardiovascular disease (CVD) are inversely associated but the mediating factors have not been totally elucidated. Our aim was to analyze the mediating role of modifiable risk factors. Cohort study using the REGICOR population cohorts. Participants without previous CVD were included (n = 9226). Marginal structural models were used to analyze the association between education and CVD incidence at 6 years of follow-up. Mediation by modifiable risk factors (diabetes, dyslipidemia, hypertension, smoking, body mass index, and physical activity) was assessed using the counterfactual framework. Participants with a university degree had a CVD incidence hazard ratio (HR) of 0.51 (95% confidence interval (CI) = 0.30, 0.85), compared to those with primary or lower education. Only hypertension, BMI, and diabetes mediated the association between education and CVD incidence, accounting for 26% of the association (13.9, 6.9, and 5.2%, respectively). Sensitivity analyses showed that hypertension was the strongest mediator (average causal mediation effect [95% CI] = increase of 2170 days free of CVD events [711, 4520]). The association between education and CVD incidence is partially mediated by hypertension, BMI, and diabetes. Interventions to decrease the prevalence of these risk factors could contribute to diminish the CVD inequalities associated with educational level.

## Introduction

There are a number of determinants that affect health. The World Health Organization has defined that determinants of health include the social and economic environment, the physical environment, and the individual characteristics and behaviors^1^. The more specific drivers are 8 including education, income, and social status. Regarding cardiovascular disease (CVD) it has been observed that the factors that have been involved in the decrease in CVD mortality, such as prevention and treatment, have not equally reached all social groups^2^. And more importantly, the opportunities to reduce morbidity and mortality due to CVD are associated to addressing the social determinants of CVD as disparities have increased over time^3^. It is therefore essential to understand the association of social determinants not only with disease incidence but also with disease risk factors as these can be targeted with specific interventions. In the last decades an inverse association between socioeconomic status (SES) and CVD has been observed^4^, and a number of studies have shown that lower SES is associated with higher risk of CVD^5–10^. However, which are the factors explaining this association is not entirely clear. Identifying the mediating factors is the first step to diminish CVD inequalities associated to different SES, particularly regarding modifiable factors, which can be tuned with prevention such as lifestyle interventions on diet, physical activity, and smoking, and pharmacological treatment.

A number of studies have analyzed the factors underlying the association between SES and CVD^11–17^. And more recently some authors have examined the mediating role of modifiable classical cardiovascular risk factors (hypertension, diabetes, and dyslipidemia) and/or lifestyle factors (smoking, physical activity -PA-, body mass index -BMI-, and diet)^18–20^. All these studies have shown evidence of the implication of both classical and lifestyle modifiable risk factors on the association between SES and CVD but their results were highly heterogeneous. In addition, these studies have not used new methodologies to estimate the effect of SES on CVD through a set of mediators and have not evaluated whether awareness, treatment, or control of classical risk factors is playing a role.

To fill this gap, the present study used a large population based cohort^21,22^ to analyze the mediating role of modifiable risk factors on the association between education, as a SES indicator, and 6-year CVD incidence.

## Results

### Descriptive analysis by education level

**Figure 1:**
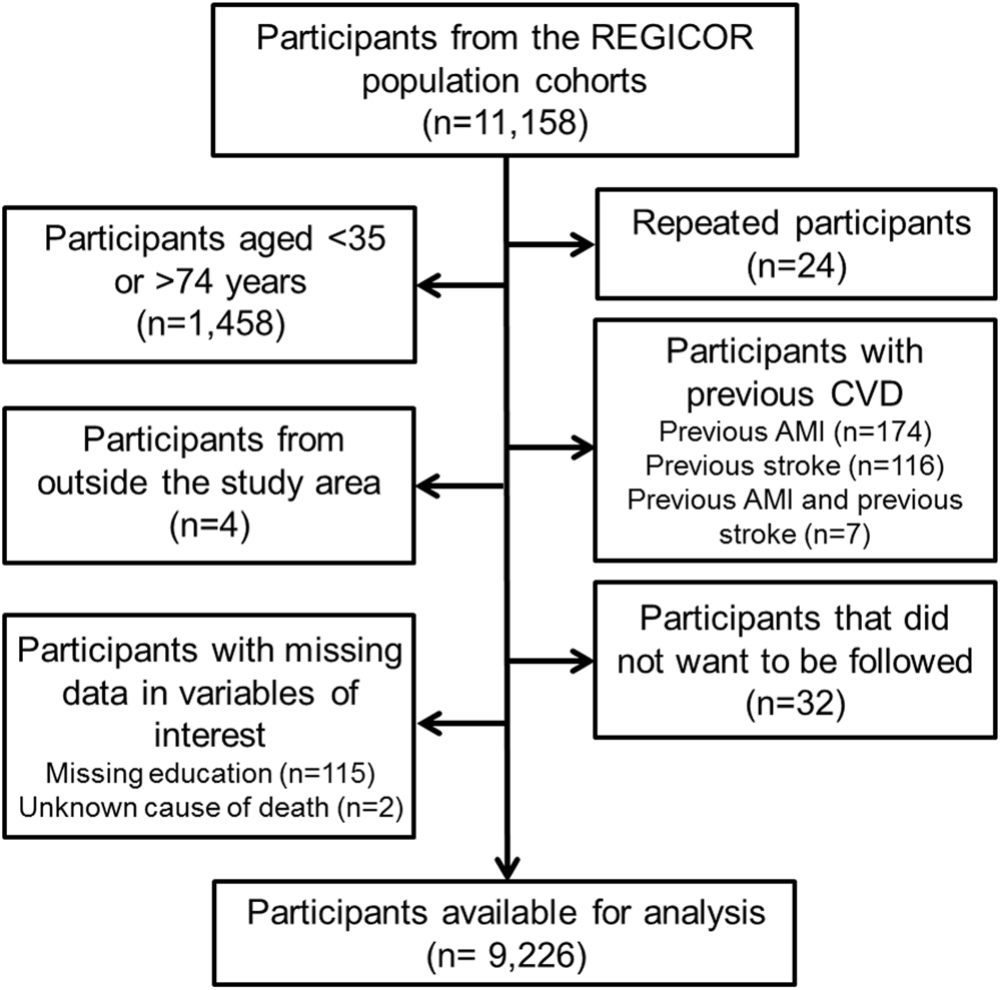
Flow chart of the participants included in the study.

From the 9226 included participants (Fig. 1), 5322 (58%) received primary (4990) or lower education (332), 2323 (25%) reached secondary education, and 1581 (17%) obtained a university degree. More educated participants were significantly younger and more frequently men, had a lower prevalence of diabetes, hypertension and dyslipidemia, and a higher prevalence of smoking (Table 1). Higher education was also associated with lower BMI, more vigorous PA practice, higher perceived physical health and with having a non-manual occupation. The percentage of participants who had a CV event in the follow-up diminished from the less to the most educated (5.7–2.9–2.2%).

**Table 1:** Characteristics of the included participants by education level.

	Elementary education	Secondary Education	University Education	p-trend	N
	N = 5,322	N = 2,323	N = 1,581		
Age (years)^a^	56.8 (10.8)	49.9 (9.9)	49.4 (9.7)	<0.001	9226
Sex (% female)	2,885 (54.2%)	1,241 (53.4%)	768 (48.6%)	<0.001	9226
Diabetes	853 (16.0%)	234 (10.1%)	129 (8.2%)	<0.001	9226
Dyslipidemia	2,426 (45.6%)	832 (35.8%)	523 (33.1%)	<0.001	9226
Hypertension	2,492 (46.8%)	677 (29.1%)	391 (24.7%)	<0.001	9226
Smoking				<0.001	9117
Non-smokers	3,138 (59.7%)	1,095 (47.5%)	700 (44.9%)		
Ex-smokers	1,053 (20.0%)	530 (23.0%)	445 (28.5%)		
Current smokers	1,063 (20.2%)	679 (29.5%)	414 (26.6%)		
BMI (kg/m^2^)^a^	28.1 (4.6)	26.6 (4.4)	25.9 (4.2)	<0.001	9151
Light PA (METs)^a^	96.8 (131)	76.2 (105)	70.2 (95.8)	<0.001	9135
Moderate PA (METs)^a^	107 (206)	88.9 (158)	89.3 (123)	<0.001	9135
Vigorous PA (METs)^a^	92.6 (203)	126 (195)	147 (215)	<0.001	9135
Total PA (METs)^a^	297 (337)	291 (290)	306 (282)	0.339	9135
Inactive	4,895 (93.0%)	2,118 (92.1%)	1,444 (91.9%)	0.196	9135
Social class (% manual)	3,948 (79.7%)	948 (41.9%)	129 (8.3%)	<0.001	8774
Quality of life – PCS^a^	48.4 (9.3)	51.6 (7.8)	53.0 (6.9)	<0.001	8500
Quality of life – MCS^a^	47.6 (11.2)	47.3 (10.5)	47.6 (10.0)	0.650	8500
Cohort				<0.001	9226
1995	1,068 (20.1%)	204 (8.8%)	66 (4.2%)		
2000	1,740 (32.7%)	497 (21.4%)	222 (14.0%)		
2005	2,514 (47.2%)	1,622 (69.8%)	1,293 (81.8%)		
Follow-up (days)^a^	3,108 (1,178)	2,754 (991)	2,493 (749)	<0.001	9226
CVD events	304 (5.7%)	67 (2.9%)	34 (2.2%)	<0.001	9226

N(%) is shown except for ^a^were mean and standard deviation is presented. P-trends were obtained with the Pearson test. BMI: body mass index; CVD: cardiovascular disease MCS: mental component score from the SF-36 questionnaire; PA: physical activity; PCS: physical component score from the SF-36 questionnaire.

### Association between education and CVD incidence

**Figure 2:**
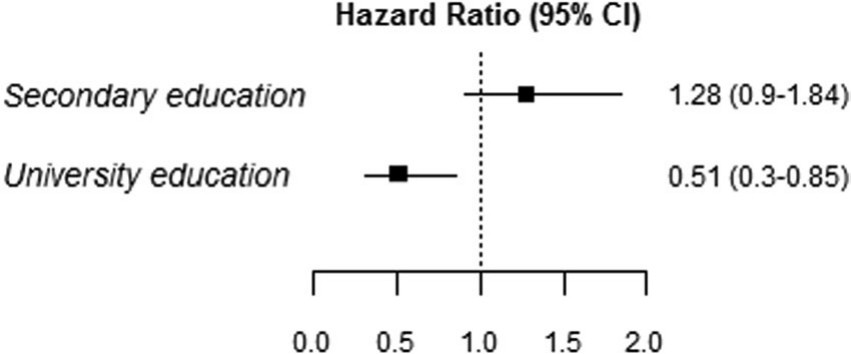
Hazard ratio (HR) and 95% confidence interval (CI) of cardiovascular disease incidence for participants with secondary and university education compared to participants with primary or lower education. The association between education and CVD incidence was analyzed with a marginal structural survival model. Inverse probability weights and robust standard errors were included in the model.

Participants who obtained a university degree had a CV event hazard ratio (HR) of 0.51 (95% confidence interval (CI): 0.30–0.85), compared to the ones who received primary or lower education (Fig. 2). No association was observed when comparing participants that completed secondary education only to the ones that received primary or lower education. There was no effect modification by gender, while there was a slight effect modification by age in participants who reached secondary education only (Suppl. Table S1, Suppl. Fig. S1).

Sensitivity analyses for exchangeability and unmeasured confounding by categorical variables showed that those were not a concern (Suppl. Results and Tables S2 and S3). Sensitivity analyses for positivity and correct model specification assumptions showed similar results (Suppl. Results and Tables S2 and S4).

### Mediation of the association between education and CVD incidence

Hypertension, BMI, and diabetes were identified as mediators of the association between education and 6-year CVD incidence (Table 2). The average causal mediation effects (ACMEs) or indirect effects shown in Table 2 are the expected difference in the number of days without a CVD event when the mediator takes the value observed in individuals with university education compared to those with primary or lower education holding education level constant. Thus, an increase of 2170 and 815 days without a CVD event was expected when comparing hypertension and diabetes prevalence of the most educated to the prevalence in the less educated. Similarly, an increase of 1080 days without a CVD event was expected when comparing mean BMI of the most educated to the value in the less educated. The total effect of education on CVD incidence was 15600 days free of CVD events and the direct effects of the mediators analyzed are presented in Suppl. Table S5. The percentage of the education-CVD incidence association mediated by the identified mediators was 26%. Hypertension, BMI, and diabetes mediated 13.9%, 6.9%, and 5.2% of the association, respectively.

**Table 2:** Average causal mediation effects of modifiable risk factors on the association between education level and cardiovascular incidence at 6 years.

Mediator	ACME (95% CI)	p-value
Diabetes	815 (262, 1660)	<0.001
Dyslipidemia	−52 (−2030, 1830)	0.97
Hypertension	2170 (711, 4520)	<0.001
Smoking	−620 (−1960, 267)	0.18
BMI	1080 (92, 3330)	<0.001
PA	232 (−169, 867)	0.26

ACMEs and their corresponding CIs are expressed as the expected difference in the number of CVD-free days when the mediator takes the value observed in individuals with university education compared to those with primary or lower education. Mediators were modeled with linear (BMI, PA), logistic (Diabetes, Dyslipidemia, Hypertension), and ordered logistic (Smoking) regression models; CVD incidence with a parametric survival model. Monte Carlo simulations were used to obtain the CI.ACME: average causal mediation effect; BMI: body mass index; CI: confidence interval;PA: physical activity.

These results were consistent in several sensitivity analyses for residual confounding and measurement errors (Suppl. Table S5). Including education-mediators interaction terms in the outcome models pointed to hypertension as the strongest mediator (Suppl. Table S5).

Awareness, treatment, and control of diabetes and hypertension in individuals with these risk factors were not playing a role in mediating the association between education and 6-year CVD incidence (Suppl. Table S6).

## Discussion

Our study showed an inverse association between education and CVD incidence in general population from North-Eastern Spain when comparing participants with university education to participants with primary or lower education. Mediation analyses identified that 26% of this association was mediated by classical and lifestyle modifiable risk factors such as diabetes, hypertension, and BMI. Further analyses showed that awareness, treatment, and control of diabetes and hypertension were not mediating the association between education and CVD incidence, and that hypertension was the strongest mediator.

Our results showed a reduction of CVD incidence in participants with university education compared to participants with primary or lower education. While the association between education and CVD incidence had not been assessed with causal methods before, our estimates are in line with others from population-based cohorts^6,9,23,24^. This result suggest that different prevention strategies or at least different intensities in prevention should be provided to individuals by educational level to account for the excess CVD risk and for the clustering of CVD risk factors in low socioeconomic groups^25^.

A graded inverse association between SES and CVD has been described in some studies^13,14^. We think that the lack of effect observed when comparing participants with secondary education with those with primary or lower education in the present analysis could be caused by a more heterogeneous secondary education group. This expectation is in accordance with the effect modification identified for age in the secondary education group. In addition, a possible birth cohort effect characterized by different percentages of the 3 educational categories in the 3 included cohorts could be playing a role.

Our results showed that diabetes, hypertension, and BMI, were mediators of the association between education and CVD incidence. These mediators accounted for 26% of the total effect of education on CVD incidence. In previous studies the effect of education on CHD incidence was attenuated up to a 70% after adjusting for CV and behavioral risk factors^6,19,24^. More recent studies focusing on the mediation of the association between education and CHD showed that 21–57% of the effect was accounted for behavioral and biological risk factors^10,18,20^. These and other studies pointed to smoking, BMI, hypertension, PA, lipid profile, and diabetes as mediators of the association between education and CVD incidence^10,11,13,15,16,20,24,26^.

From the 6 modifiable risk factors analyzed (diabetes, dyslipidemia, hypertension, smoking, BMI, and PA) only diabetes, hypertension, and BMI, were identified as mediators of the association between education and CVD incidence. There are no studies analyzing the mediators of this association using the counterfactual framework, however, a recent study by Kershaw *et al*., which estimates indirect effects of classical and lifestyle risk factors simultaneously may be a fair comparison^20^. In this study, in addition to diabetes, hypertension, and BMI, they found that hypercholesterolemia, smoking, and PA were also mediators of the education-CHD relationship. Disagreement on the mediating effect of hypercholesterolemia and PA is probably influenced by differences in variable measurement. In the REGICOR cohorts PA was based on the Minnesota leisure-time physical activity questionnaire, cholesterol was measured in fasting samples and dyslipidemia was based on LDL-C levels. While Kershaw *et al*. obtained the PA information from a single question, measured cholesterol in non-fasting samples and based hypercholesterolemia in total cholesterol levels.

Our results did not show a significant mediation effect of smoking in the association between education and CVD incidence in contrast to the literature and to previous studies of mediation in this topic^18–20^. This lack of effect is probably influenced by several factors being probably the more important the differential effect of smoking on the association between education and CVD incidence among European populations. In a study of European cohorts it was found that the percentage change in the relative index of inequality regarding education for CHD incidence due to smoking differed between European countries^10^. This difference was observed in both men and women, and in women it showed a contrary sign in Southern and in Northern European populations.

The identification of body mass index, diabetes, and particularly hypertension, as mediators of the education-CVD association, point to these risk factors as potential targets for interventions to reduce CVD in low socioeconomic groups. While there is a scarcity of interventions to improve risk factor control in those with low literacy^2^ the few existing studies show positive effects. An American program developed individual- and community-level approaches targeting CVD health behaviors for blacks in their communities^27^. The program was associated with significant improvements in diet among others determinants^28^. In addition, in a study from Iran it was found that an educational and environmental program was able to reduce adiposity measures in low-literate women^29^. As these examples suggest, current recommendations are to include a combination of both population- and individual-level strategies to lower CVD risk and to target high-risk individuals^30^.

While diabetes and hypertension had already been described as mediators of the education-CVD incidence association, no study had previously analyzed the robustness of this effect regarding the causal mediation assumptions, or the effect of awareness, treatment, and control of these risk factors. Our results showed that hypertension was the most robust mediator based on the effect of the education-risk factor interaction terms, which yielded almost equal estimates for the hypertension ACME. In addition, neither awareness, treatment, or control, mediated the education-CVD incidence association in participants with diabetes or hypertension. Although the ACMEs for diabetes awareness and treatment, and for hypertension awareness were close to significant. It is possible that the reduced number of individuals with diabetes or hypertension, 13% and 42%, respectively, was an insufficient sample size to examine the mediation effect of diabetes and hypertension awareness, treatment, and control.

Modifiable CV risk factors explained only 26% of the association between education and CVD incidence. We expect that other factors would explain part of the remaining effect. Diet could also act as a mediator on this association, but based on previous studies we believe this effect would be small once other modifiable risk factors have been accounted for^20^. Other mediators could be biological (e.g. stress biomarkers), material (e.g. financial issues), early life and psychological characteristics (e.g. material and cognitive childhood factors), or socio-economic, cultural or environmental factors as pointed out by some authors^12,14,31,32^. Psychological mechanisms are probably accounting for a part of the non-explained association between education and CVD as a large number of studies have described a strong relation between emotional states and CVD risk, particularly depression^33^. However, the scarcity of studies examining the mediating role of psychological factors precludes the estimation of this effect.

To our knowledge, this is the first study estimating ACMEs of multiple modifiable risk factors in the education-CVD incidence association. Our study is also the first addressing the role of modifiable risk factor awareness, treatment, and control. The main strengths are the use of a large population-based cohort (n = 9,226), randomly selected from a reference population of 600,000 inhabitants, and novel statistical tools such as the counterfactual framework.

There are some limitations that should be considered though. First, due to limitations on the available analytical tools we could not assess the confounding effect of some mediators (e.g. BMI) on the effect of other mediators (e.g. diabetes and hypertension). Another limitation is the assumption of no unmeasured confounding. While this assumption cannot be tested, its plausibility can be assessed with sensitivity analyses. Our sensitivity analyses showed that unmeasured confounding seem not to be affecting the estimates. Finally, we did not include medication adherence and other socioeconomic factors which could explain part of the association between education and CVD incidence and affect diabetes and hypertension control as they were not available in the REGICOR cohorts.

In conclusion, individuals from North-Eastern Spain with a university degree had a CV event HR of 0.51 (95% CI = 0.30–0.85) at 6 years, compared to the ones who received primary or lower education. The association between education and CVD incidence was partially mediated by diabetes, hypertension, and BMI, being hypertension the strongest mediator. These mediating factors, particularly, hypertension, could be tackled to diminish CVD risk among population with a low education level.

## Methods

### Study population

The Registre Gironí del Cor (REGICOR) cohorts, were recruited in three population-based surveys conducted in the province of Girona (north-eastern Spain) in 1995 (n = 1748), 2000 (n = 3058) and 2005 (n = 6352)^21^. At baseline, participants underwent a physical examination and completed interview-administered questionnaires^22^. Participants were followed for cardiovascular (CV) events until 2011–2013 (median follow-up 7 years). This study included participants from the REGICOR cohorts aged 35–74 years with no history of CVD (acute myocardial infarction -AMI- or stroke), and with available data in the variables of interest (n = 9226) (Fig. 1).

REGICOR surveys were approved by the Parc de Salut Mar ethics committee and informed consent was obtained from all participants. All experiments were performed according to guidelines and regulations.

### Exposure and outcome

The exposure under study was education level attained, reported by the participants at baseline. Education was categorized in three levels: primary or lower education (any education below the secondary school diploma), secondary (secondary school diploma or higher without a college degree) and university (college degree).

The outcome of interest was CVD incidence during the first 6 years of follow-up analyzed as a time-to-event variable. CVD events were identified by participant interviews, medical record revision, and data linkage with the REGICOR AMI population registry^34^ and the official mortality register. The REGICOR AMI registry registered all AMI that occurred in 1977–2009 in the same region were the population cohorts were recruited. Fatal events were identified using ICD9 codes 410–414 and ICD10 codes I20-I25 for coronary events, and ICD9 codes 430–438 and ICD10 codes I60-I69 for cerebrovascular events. Non-fatal cases were identified from medical records and physician notes, and were classified in committee according to standardized criteria: i) AMI was defined according to symptoms, electrocardiogram, and biomarkers of necrosis; ii) angina was defined according to the presence of symptoms and objective demonstration of ischemia on ECG or presence of coronary stenosis; iii) coronary revascularization, including percutaneous invasive revascularization and surgery; iv) stroke was defined based on objective evidence of cerebral ischemic injury (pathological, imaging, or other) in a defined vascular distribution, or clinical evidence of cerebral focal ischemic injury based on symptoms persisting ≥24 hours or until death, and other etiologies excluded^35^.

### Covariates

Descriptive analyses by education level included sociodemographic variables (age and gender), CV risk factors (diabetes, hypertension, dyslipidemia, smoking, BMI, and PA), social class, and quality of life. CV risk factors were recorded at baseline as previously described^21,22^. Presence of diabetes, hypertension, and dyslipidemia were based on previous diagnosis, current treatment and baseline values of fasting glucose, blood pressure and low density lipoprotein cholesterol (LDL-C). The cutoff for fasting glucose was ≥126 mg/dL, for systolic and diastolic blood pressure was ≥140 and ≥90 mmHg, respectively, and for LDL-C was ≥160 mg/dL. Among participants with diabetes or hypertension we also analyzed the awareness (proportion who declared to be aware of having the risk factor), treatment (proportion treated), and control (proportion with glucose or blood pressure under the defined cutoffs). Smoking was categorized in non-smokers, ex-smokers (more than 1 year), and smokers (at least 1 cigarette/day). We calculated BMI as weight divided by squared height (kg/m^2^). Individuals were classified as inactive if they spent <600 metabolic equivalents (METs)/week of moderate PA and <400 METs/week of vigorous PA, and as active if they did ≥600 METs/week of moderate PA or ≥400 METs/week of vigorous PA^36^, based on the Minnesota leisure-time physical activity questionnaire. Social class was categorized in manual and non-manual based on occupation as recommended by the Spanish Society of Epidemiology^37^. Quality of life physical and mental summary components were obtained from the Spanish version of the SF-36 Health survey^38^.

### Statistical analysis

A directed acyclic graph for the causal association of education and CVD was created using DAGitty^39^ based on previous research experience and literature evidence (Suppl. Fig. 2).

Continuous variables were summarized as mean and standard deviation, and categorical variables as proportions. P-value for trend was computed using the Pearson test.

The association between education and CVD incidence was examined with a marginal structural survival model for the outcome time to CVD event in the first 6 years of follow-up, using inverse-probability-of-treatment-and-censoring (IPTC) weights and robust standard errors. The proportional hazard assumption was confirmed graphically and by the nonzero slope test. IPTC weights were calculated as the product of stabilized exposure of interest (education level) and censoring weights^40^. For exposure weights, the denominator was each participant’s probability of having their education level based on all measured pre-education covariates (age, gender and cohort), while the numerator was this probability based on the intercept and no covariates. Age was included as a smoothing spline using a penalized spline basis. Cohort was included as a 3 category variable. Multinomial logistic regression was used to create the weight models for exposure. For censoring weights, the denominator was each participant’s probability of being censored based on age, gender, cohort and education, while the numerator was this probability based on education only. Logistic regression was used to create the weight models for censoring. Effect modification by age and gender was analyzed by adding these covariates plus their interaction with education to the structural model.

Assumptions of the marginal structural model were analyzed as follows^41^. Exchangeability was examined by analyzing the effect of unmeasured categorical confounders. First, the inclusion of place of birth in the weights was analyzed. Place of birth was included as a 3 category variable (Catalonia, north region of the rest of Spain, and south region of the rest of Spain). Second, the obsSens package from R was used to analyze potential unknown confounders. To analyze positivity, we re-fitted the models excluding the subgroup were there was a low number of participants with university education (age > 70 years). Model specification was assessed by using different models (survival and logistic), by excluding participants from cohort 2000, and by redoing the analysis with different follow-up times (4, 5, 7 and 8 years).

Mediation of the association between education level and 6-year CVD incidence by modifiable risk factors (diabetes, hypertension, dyslipidemia, smoking, BMI, and PA) was analyzed with the counterfactual framework using the R package mediation^42^. The mediation effect of awareness, treatment, and control of the significant treatable mediators was also examined in individuals with each of the risk factors. The counterfactual framework allows the estimation of indirect effects of a treatment on an outcome through a set of mediators based on models for the mediators and the outcome. Mediator models were linear (BMI, and PA), logistic (diabetes, dyslipidemia, and hypertension), or ordered logistic (smoking) depending on the mediator. The outcome (6-year CVD incidence) was modeled using a parametric survival model. Quasi-bayesian confidence intervals were calculated for the estimates using 1000 simulations. Age, gender and cohort were included as pre-treatment confounders. We calculated the percentage of mediated association as the risk factor average causal mediation effect (indirect effect) divided by the total effect of education on CVD incidence^20^.

Assumptions of the mediation analysis were examined as follows^43^. The assumption of temporal ordering was satisfied as the exposure (education level attained prior to baseline) preceded the outcome (CVD incidence during follow-up), mediators –measured at baseline- preceded the outcome –measured from baseline on-, and exposure preceded the mediators. Unmeasured confounding was examined with a sensitivity analysis which included place of birth and social class as confounders in the mediator and outcome models. Measurement error of the mediators was assessed for PA by treating this variable as continuous and categorical in separate models. Estimate bias due to interaction between exposure and mediators was assessed in sensitivity analysis by including this interaction in the outcome models and checking the change in the indirect effect estimates.

Analyses were conducted in R statistical software version 3.2.3^44^.

### Data Availability

The dataset analyzed during the current study is available from the corresponding authors on reasonable request.

## Electronic supplementary material

Supplementary data
