# Supplementary material for: The association between education and cardiovascular disease incidence is mediated by hypertension, diabetes, and body mass index
Source: Sci Rep. 2017 Sep 28;7:12370. doi: 10.1038/s41598-017-10775-3 (PMC5620039; doi:10.1038/s41598-017-10775-3)
Supplement: Supplementary file 1 — Supplementary data [file 41598_2017_10775_MOESM1_ESM.doc]

**SUPPLEMENTARY INFORMATION**

**The association between education and cardiovascular disease incidence is mediated by hypertension, diabetes, and body mass index.**

Irene R Dégano, Jaume Marrugat, Maria Grau, Betlem Salvador-González, Rafel Ramos, Alberto Zamora, Ruth Martí, Roberto Elosua

**SUPPLEMENTARY RESULTS**

**Assumptions of the structural model**

Sensitivity analyses for exchangeability are presented in Suppl. Tables S2 and S3. When place of birth was included in the weights similar results were obtained but with a larger effect size (HR=0.41, 95% CI: 0.24-0.69) (Suppl. Table S2, Model specification (MS) =2). Under a plausible prevalence range among education groups and assuming the minimum and maximum effect on CVD, an unmeasured categorical confounder would not modify significantly the main effect in 75% of the scenarios (HR=0.32-0.59 and 95% CI below 1) (Suppl. Table S3). In the other 25% the estimate was <1 but the CI included 1.

Structural violation of positivity was not expected in our study. Random violation of positivity was not present when the number of participants by education level was analyzed by the main confounders. However, a lower number of participants with university education was observed particularly in those aged 70-74 years from cohorts 1995 and 2000. Sensitivity analysis excluding these participants did not modify significantly the effect estimate (Suppl. Table S2, MS 3-4).

Sensitivity analysis for model specification showed similar results when using logistic regression models (Suppl. Table S4) and when time of follow-up was modified (Suppl. Table S2, MS 6-9). When participants from cohort 2000 were excluded, having university education was associated with lower incidence of CV events although the effect size decreased and the CI widened (HR=0.65, 95% CI: 0.39-1.08) (Suppl. Table S2, MS 5).

**SUPPLEMENTARY TABLES AND FIGURES**

**Supplementary Table S1.** Effect modification by age and sex.

|  | Education level | Parameter estimate (95% CI) |
| --- | --- | --- |
| Age | High school education  University education | **1.04 (1.00, 1.07)**  1.02 (0.98, 1.06) |
| Sex | High school education  University education | 0.65 (0.30, 1.40)  0.77 (0.23, 2.52) |

The estimate and 95% CI presented correspond to the estimate of the interaction terms between age,and education (taking primary or lower education as reference), and between sex and education (taking men and primary or lower education as reference) .

CI: confidence interval

**Supplementary Table S2.** Sensitivity analyses for the structural model assumptions on exchangeability, positivity and model specification.

|  | **Weights** | | | **Effect on CVD incidence** | | |
| --- | --- | --- | --- | --- | --- | --- |
| **MS** | Mean | sd | range | estimate | Robust se | Robust CI |
| **1** | **1.00** | **0.70** | **0.41/15.37** | **0.51** | **0.26** | **0.30, 0.85** |
| *Exchangeability* | | | | | | |
| 2 | 1.00 | 0.83 | 0.32/17.72 | 0.41 | 0.27 | 0.24, 0.69 |
| *Positivity* | | | | | | |
| 3 | 1.00 | 0.70 | 0.45/20.09 | 0.51 | 0.30 | 0.28, 0.93 |
| 4 | 1.00 | 0.65 | 0.43/14.99 | 0.59 | 0.26 | 0.35, 0.99 |
| *Model specification* | | | | | | |
| 5 | 0.99 | 0.75 | 0.48/20.20 | 0.65 | 0.26 | 0.39, 1.08 |
| 6 | 4 years of follow-up | | | 0.48 | 0.31 | 0.26, 0.89 |
| 7 | 5 years of follow-up | | | 0.46 | 0.27 | 0.27, 0.78 |
| 8 | 7 years of follow-up | | | 0.58 | 0.24 | 0.37, 0.93 |
| 9 | 8 years of follow-up | | | 0.59 | 0.23 | 0.38, 0.94 |

CI: confidence interval; MS: model specification; sd: standard deviation; se: standard error.

1: final model, weights with age, sex and cohort;

2: same as 1 but including place of birth in the weights;

3: weights as in 1, but excluding participants aged >69 years (n=8,322);

4: weights as in 1, but excluding participants aged >69 years from cohorts 1995 and 2000 (n=8,888);

5: weights as in 1, but excluding participants from cohort 2000 (n=6,774);

6-9: weights as in 1, but modifying the time of follow-up. In these MS, the weight mean, sd and range are not shown as they are the same as the ones for the final model (MS=1).

**Supplementary Table S3.** Sensitivity analyses for the structural model assumption on unmeasured categorical confounders.

Panel A: Effect estimate of the confounder on CVD incidence = 0.5

|  | 0.1 | 0.2 | 0.3 | 0.4 | 0.5 | 0.6 | 0.7 |
| --- | --- | --- | --- | --- | --- | --- | --- |
| 0.1 | 0.508  (0.303,0.853) | 0.481  (0.287,0.808) | 0.455  (0.271,0.763) | 0.428  (0.255,0.718) | 0.401  (0.239,0.673) | 0.374 (0.223,0.628) | 0.348  (0.207,0.584) |
| 0.2 | 0.536  (0.320,0.900) | 0.508  (0.303,0.853) | 0.480  (0.286,0.805) | 0.452  (0.269,0.758) | 0.423  (0.252,0.711) | 0.395 (0.235,0.663) | 0.367  (0.219,0.616) |
| 0.3 | 0.568  (0.338,0.953) | 0.538  (0.321,0.903) | 0.508  (0.303,0.853) | 0.478  (0.285,0.803) | 0.448  (0.267,0.752) | 0.418  (0.249,0.702) | 0.389  (0.232,0.652) |
| 0.4 | **0.603**  **(0.360,1.013)** | 0.572  (0.341,0.959) | 0.540  (0.322,0.906) | 0.508  (0.303,0.853) | 0.476  (0.284,0.800) | 0.445  (0.265,0.746) | 0.413  (0.246,0.693) |
| 0.5 | **0.644**  **(0.384,1.080)** | **0.610**  **(0.363,1.023)** | 0.576  (0.343,0.967) | 0.542  (0.323,0.910) | 0.508  (0.303,0.853) | 0.474  (0.283,0.796) | 0.440  (0.262,0.739) |
| 0.6 | **0.690**  **(0.411,1.157)** | **0.653**  **(0.389,1.096)** | **0.617**  **(0.368,1.036)** | 0.581  (0.346,0.975) | 0.544  (0.324,0.914) | 0.508  (0.303,0.853) | 0.472  (0.281,0.792) |
| 0.7 | **0.743**  **(0.443,1.246)** | **0.704**  **(0.419,1.181)** | **0.664**  **(0.396,1.115)** | **0.625**  **(0.373,1.050)** | 0.586  (0.349,0.984) | 0.547  (0.326,0.918) | 0.508  (0.303,0.853) |

Panel B: Effect estimate of the confounder on CVD incidence = 2.1

|  | 0.1 | 0.2 | 0.3 | 0.4 | 0.5 | 0.6 | 0.7 |
| --- | --- | --- | --- | --- | --- | --- | --- |
| 0.1 | 0.508  (0.303,0.853) | 0.559  (0.333,0.937) | **0.609**  **(0.363,1.022)** | **0.659**  **(0.393,1.106)** | **0.710**  **(0.423,1.191)** | **0.760 (0.453,1.275)** | **0.810**  **(0.483,1.360)** |
| 0.2 | 0.462  (0.275,0.776) | 0.508  (0.303,0.853) | 0.554  (0.330,0.930) | **0.600**  **(0.357,1.007)** | **0.646**  **(0.385,1.084)** | **0.691 (0.412,1.160)** | **0.737**  **(0.439,1.237)** |
| 0.3 | 0.424  (0.253,0.712) | 0.466  (0.278,0.782) | 0.508  (0.303,0.853) | 0.550  (0.328,0.923) | 0.592  (0.353,0.994) | **0.634**  **(0.378,1.064)** | **0.676**  **(0.403,1.135)** |
| 0.4 | 0.392  (0.233,0.657) | 0.431  (0.257,0.723) | 0.469  (0.280,0.788) | 0.508  (0.303,0.853) | 0.547  (0.326,0.918) | 0.586  (0.349,0.983) | **0.625**  **(0.372,1.048)** |
| 0.5 | 0.364  (0.217,0.611) | 0.400  (0.238,0.671) | 0.436  (0.260,0.732) | 0.472  (0.281,0.792) | 0.508  (0.303,0.853) | 0.544  (0.324,0.913) | 0.580  (0.346,0.974) |
| 0.6 | 0.340  (0.202,0.570) | 0.373  (0.223,0.627) | 0.407  (0.243,0.683) | 0.441  (0.263,0.740) | 0.474  (0.283,0.796) | 0.508  (0.303,0.853) | 0.542  (0.323,0.909) |
| 0.7 | 0.319  (0.190,0.535) | 0.350  (0.209,0.588) | 0.382  (0.228,0.641) | 0.413  (0.246,0.694) | 0.445  (0.265,0.747) | 0.477  (0.284,0.800) | 0.508  (0.303,0.853) |

Columns represent the range of expected prevalence that would have the unmeasured confounder in the participants that received elementary education. Rows represent the range of expected prevalence that would have the unmeasured confounder in the participants that obtained a university degree. The prevalence range was assumed from the observed range in CV risk factors across education levels. Analysis were performed for the maximum expected effect in decreasing CVD incidence (Panel A) and for the maximum expected effect in increasing CVD incidence (Panel B) based on the age- and sex- adjusted effects of the other CV risk factors. CV: cardiovascular; CVD: cardiovascular disease.

**Supplementary Table S4.** Sensitivity analyses on model specification: estimates by logistic regression.

| Education level | OR (95% CI) |
| --- | --- |
| High school education  University education | 1.28 (0.92, 1.77)  0.50 (0.30, 0.84) |

As in the main analysis the reference category was elementary education.

CI: confidence interval; OR: odds ratio

**Supplementary Table S5.**  Average direct mediation effects of modifiable risk factors on the association between education level and cardiovascular incidence at 6 years.

| Mediator | ADE (95% CI) | p-value |
| --- | --- | --- |
| Diabetes | 10900 (-9940, 39800) | 0.37 |
| Dyslipidemia | 14300 (-11000, 45900) | 0.31 |
| Hypertension | 11800 (-12300, 43700) | 0.38 |
| Smoking | 12600 (-10100, 44400) | 0.34 |
| BMI | 20100 (-27000, 89400) | 0.41 |
| PA | 12900 (-9410, 42000) | 0.27 |

Mediators were modeled with linear (BMI, PA), logistic (Diabetes, Dyslipidemia, Hypertension), and ordered logistic (Smoking) regression models; CVD incidence with a parametric survival model. Monte Carlo simulations were used to obtain the CI.ADE: average direct effect; BMI: body mass index; CI: confidence interval; PA: physical activity.

**Supplementary Table S6.** Average causal mediation effects of modifiable risk factors on the association between education level and cardiovascular incidence at 6 years, sensitivity analyses.

| Mediator | ACME (95% CI) | p-value |
| --- | --- | --- |
| Residual confounding* | | |
| Diabetes | 865 (241, 1850) | <0.001 |
| Dyslipidemia | -1550 (-4780, 4360) | 0.12 |
| Hypertension | 1590 (395, 3770) | <0.001 |
| Smoking | -766 (-2280, 239) | 0.16 |
| BMI | 2220 (218, 5520) | 0.03 |
| PA | 152 (-347, 880) | 0.57 |
| Measurement error for PA | | |
| Light PA | 65 (-400, 53) | 0.74 |
| Moderate PA | -375 (-1080, 52) | 0.09 |
| Vigorous PA | 833 (-449, 2640) | 0.25 |
| Total PA | 227 (-122, 810) | 0.27 |
| Inactive | 68 (-188, 485) | 0.68 |
| Interaction effect** |  |  |
| Diabetes | 243 (-1230, 1390) | 0.60 |
| Dyslipidemia | -40 (-1700, 1900) | 0.95 |
| Hypertension | 2760 (7472, 6720) | 0.01 |
| Smoking | -1380 (-4960, 322) | 0.14 |
| BMI | 4290 (-717, 11700) | 0.10 |
| PA | 213 (-488, 1170) | 0.54 |

* Including social class and place of birth as confounders in the mediator and outcome models.

** Including the interaction of education and each mediator in each outcome model. The difference in the ACMEs comparing the model with and without the interaction term was not significant for any mediator.

ACME: average causal mediation effect; BMI: body mass index; PA: physical activity.

**Supplementary Table S7. Average causal mediation effects of diabetes and hypertension awareness, treatment, and control, on the association between education level and cardiovascular incidence at 6 years.**

| Mediator | ACME (95% CI) | p-value |
| --- | --- | --- |
| Diabetes awareness | 1500 (-153, 5680) | 0.11 |
| Diabetes treatment | 57 (-145, 2200) | 0.15 |
| Diabetes control | 227 (-549, 1900) | 0.74 |
| Hypertension awareness | -334 (-1190, 192) | 0.22 |
| Hypertension treatment | -212 (-949, 317) | 0.42 |
| Hypertension control | 66 (-282, 543) | 0.78 |

ACME: average causal mediation effect

**Supplementary Figure 1.** Cumulative incidence of cardiovascular disease stratified by educational level and sex and age group. Line colors represent educational levels. Black, primary or lower education; grey, secondary education; red, university education.


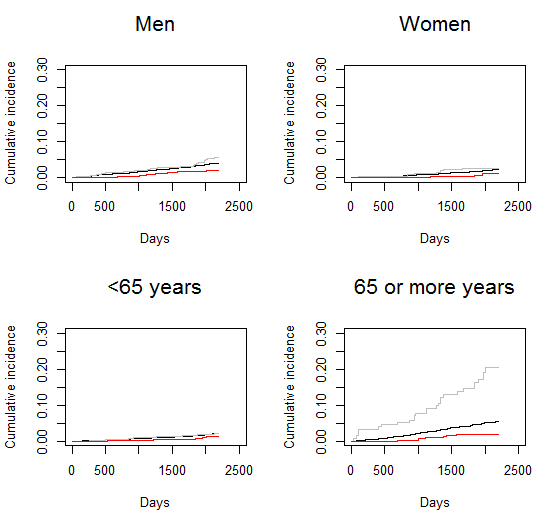


**Supplementary Figure 2.** Directed acyclic graph of the assumed relationships between variables in the path from education to cardiovascular disease (CVD) incidence. Education is the exposure and CVD is the outcome. Variables with red circles represent ancestors of both exposure and outcome. Variables with blue circles represent ancestors of the outcome. Variables with grey circles represent unobserved variables. Red and green arrows represent biasing and causal paths, respectively. Baseline_char: baseline characteristics, health_interv: health interventions,
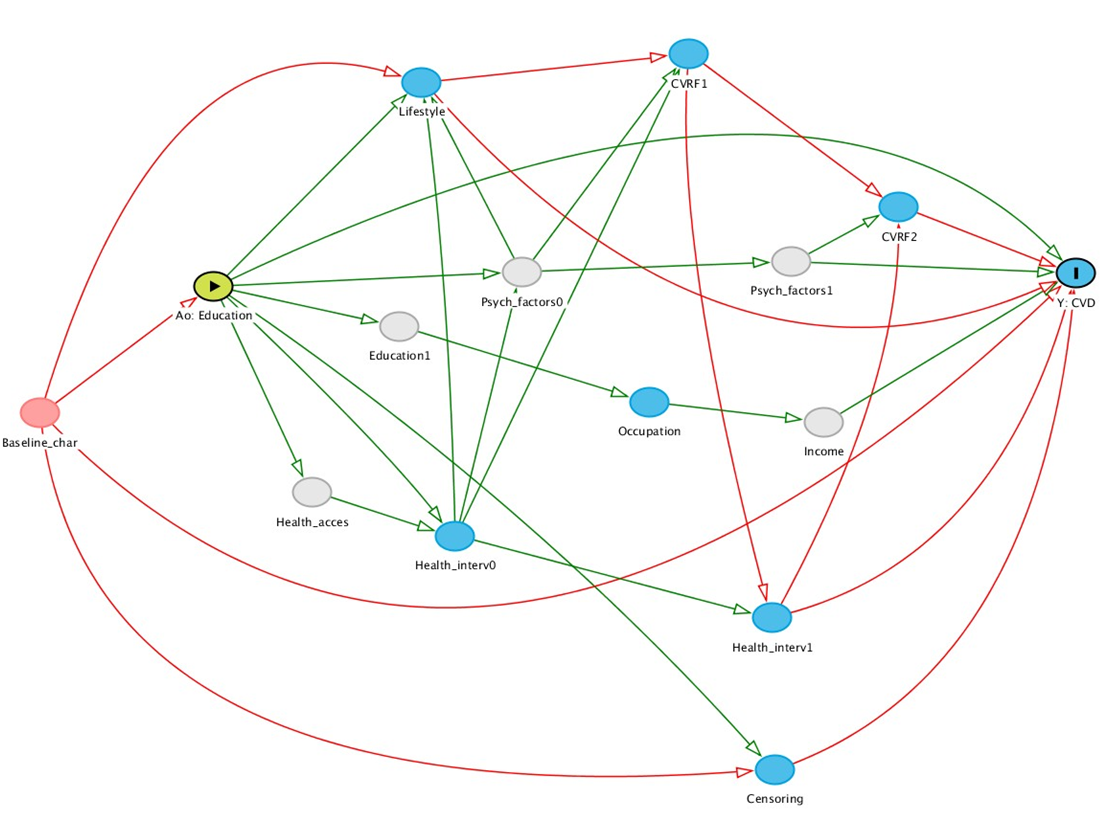
psych_factors: psychosocial factors, CVRF: cardiovascular risk factors.
